# Clinical characteristic–assisted surgical benefit stratification for resection of primary tumor in patients with advanced primary malignant bone neoplasms: a population-based propensity score–matched analysis

**DOI:** 10.3389/fonc.2023.960502

**Published:** 2023-09-06

**Authors:** Yuexin Tong, Liming Jiang, Yuekai Cui, Yangwei Pi, Yan Gong, Dongxu Zhao

**Affiliations:** ^1^ Department of Orthopedics, The China-Japan Union Hospital of Jilin University, Changchun, Jilin, China; ^2^ The Second Clinical Medical School of The Wenzhou Medical University, Wenzhou, Zhejiang, China

**Keywords:** primary malignant bone neoplasms, primary tumor resection, nomogram, survival benefit, SEER database

## Abstract

**Background:**

Primary tumor resection (PTR) is the standard treatment for patients with primary malignant bone neoplasms (PMBNs). However, it remains unclear whether patients with advanced PMBNs still benefit from PTR. This study aimed to develop a prediction model to estimate the beneficial probability of PTR for this population.

**Methods:**

This study extracted data from patients diagnosed with advanced PMBNs, as recorded in the Surveillance, Epidemiology, and End Results (SEER) database, with the period from 2004 to 2015. The patient cohort was then bifurcated into two groups: those who underwent surgical procedures and the non-surgery group. Propensity score matching (PSM) was utilized to mitigate any confounding factors in the study. The survival rates of patients from both the surgical and non-surgery groups were evaluated using Kaplan–Meier (K-M) curves analysis. Moreover, the study used this method to assess the capacity of the nomogram to distinguish patients likely to derive benefits from surgical intervention. The study was grounded in the hypothesis that patients who underwent PTR and survived beyond the median overall survival (OS) time would potentially benefit from the surgery. Subsequently, logistic regression analysis was performed to ascertain significant predictors, facilitating the development of a nomogram. This nomogram was subjected to both internal and external validation using receiver operating characteristic curves, area under the curve analysis, calibration plots, and decision curve analysis.

**Results:**

The SEER database provided a total of 839 eligible patients for the study, among which 536 (63.9%) underwent PTR. Following a 2:1 PSM analysis, patients were classified into two groups: 364 patients in the surgery group and 182 patients in the non-surgery group. Both K-M curves and multivariate Cox regression analysis revealed that patients who received PTR had a longer survival duration, observed both before and after PSM. Crucial factors such as age, M stage, and tumor size were identified to be significantly correlated with surgical benefits in patients with advanced PMBNs. Subsequently, a nomogram was developed that uses these independent predictors. The validation of this predictive model confirmed its high accuracy and excellent discrimination ability of the nomogram to distinguish patients who would most likely benefit from surgical intervention.

**Conclusion:**

In this study, we devised a user-friendly nomogram to forecast the likehood of surgical benefits for patients diagnosed with advanced PMBNs. This tool facilitates the identification of the most suitable candidates for PTR, thus promoting more discerning and effective use of surgical intervention in this patient population.

## Introduction

Primary malignant bone neoplasms (PMBNs) diverge from other forms of cancer, representing a rare category of mesenchymal-derived tumors that makes up only 0.2%–1% of all malignant tumors (1, 2). The most prevalent type of PMBNs is osteosarcoma, followed by chondrosarcoma, Ewing sarcoma, and chordoma, respectively (3). Typically, the first symptoms that patients present with at the initial diagnosis of PMBNs are pain and localized mass. Radiographic findings generally show mixed osteolytic and osteogenic aggressive bone destruction, substantial unmineralized soft tissue mass, and even pathological fractures (4, 5). In recent years, advancements in surgical techniques and adjuvant therapy have improved survival rates; the 5-year survival rate for patients with early-stage PMBNs can reach as high as 70% (6, 7). However, the prognosis remains grim for those with advanced PMBNs, particularly for patients with distant metastases (DMs). The reported 5-year survival rates for patients with metastatic osteosarcoma, Ewing’s sarcoma, and chondrosarcoma were significantly low at 25%, <30%, and 28.4%, respectively (8, 9).

Surgical intervention is generally recognized as a fundamental treatment approach for patients with PMBNs. However, for those diagnosed with advanced and metastatic disease, systemic chemotherapy, targeted molecular therapy, and palliative treatment serve as principal strategies (10–12). Emerging evidence suggests that primary tumor resection (PTR) also offers significant survival benefits for patients with advanced PMBNs (13–15). This is presumably due to the ability of PTR to counteract the immunosuppressive effects, even in the advanced stage of the disease (16). Moreover, by reducing the tumor burden *in vivo*, PTR may enhance the efficacy of postoperative chemotherapy, thus improving patient survival results (17). Notwithstanding, there are conflicting findings in the literature. For instance, a study by Song et al. reached seemingly contradictory conclusions in a study in which found that PTR was not associated with extended survival in patients with metastatic chondrosarcoma characterized by a dedifferentiated subtype and histological grade III (18). Similarly, Matsuoka and colleagues found that performing PTR did not have a positive impact on survival rates for patients with advanced vertebral column bone sarcomas (19). These findings suggest that not all patients with advanced PMBNs benefit from surgical intervention at the primary site. In addition, considerable surgical resection of the tumor or extremity can cause significant physical alternations, including disabilities and noticeable changes in appearance (20). Such changes can result in various psychiatric conditions, such as depression and anxiety, and may even contribute to an increased suicide rate (21).

Given these considerations, it is of substantial interest to explore the factors associated with surgical benefits in patients with advanced PMBNs and to create a validated instrument to assess the probability of benefit from PTR in this population. This tool could facilitate the selection of valuable PTR treatment for suitable patients and allow appropriate treatment options for frail patients. To meet this need, our study aims to construct a predictive model by analyzing data from a population-based database. This model will quantify the surgical benefit for patients with advanced PMBNs and help to identify optimal candidates for PTR.

## Methods

### Study population

**Figure 1:**
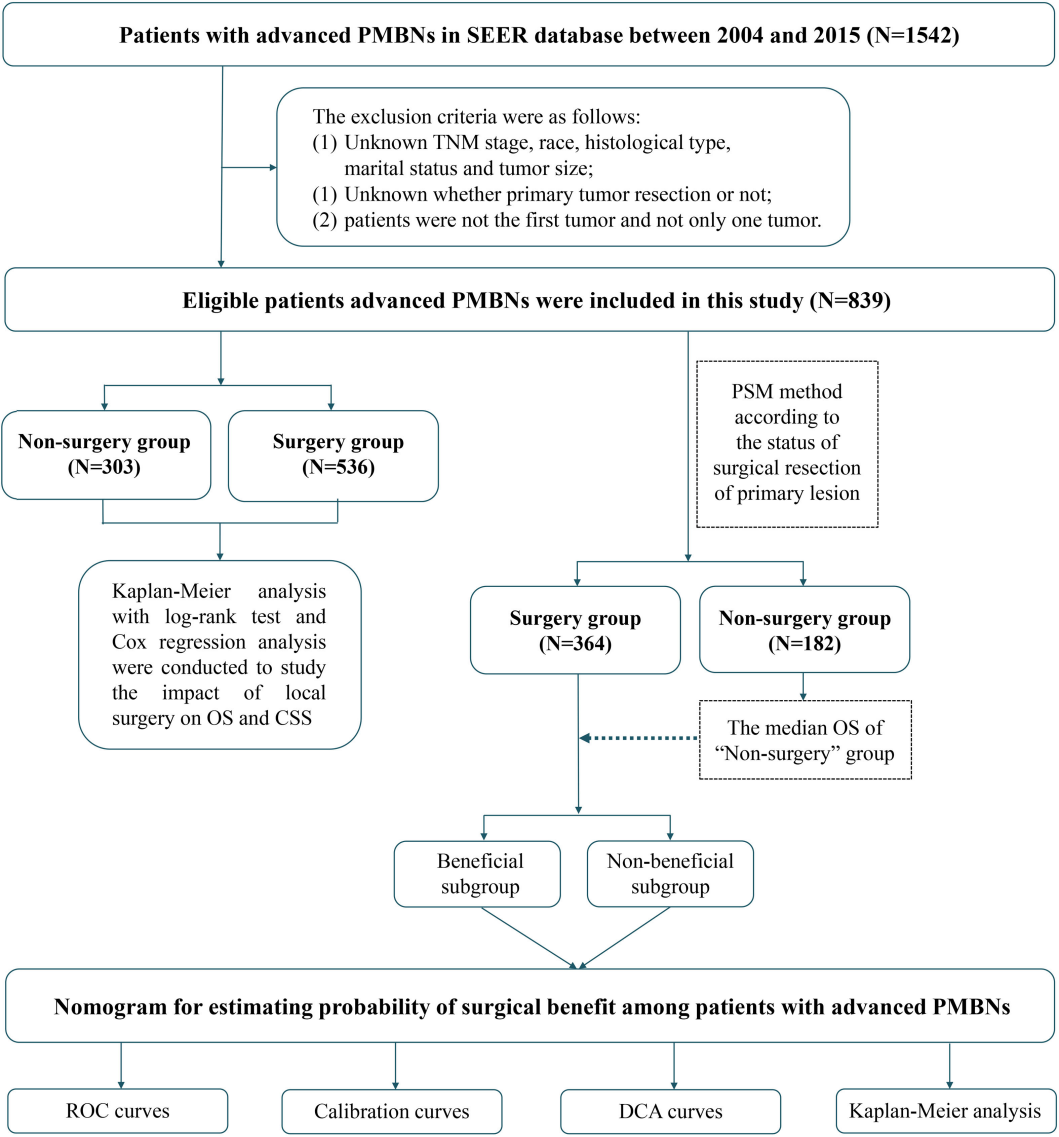
Overall flowchart of this study. SEER, Surveillance, Epidemiology, and End Results; ROC, receiver operating characteristic curve; DCA, decision curve analyses.

The Surveillance, Epidemiology, and End Results (SEER) database is the most extensive population-based cancer database, covering approximately 30% of the population in the United states (22). We have applied for access to the data released from the SEER database (SEER ID: 15685-Nov2020) and downloaded the data for patients with the field of “Site recode ICD-O-3/WHO 2008” with bone and joint during the period from 2004 to 2015 through the SEER*Stat 8.4.0 software. Furthermore, the data for the external validation set were obtained from the China–Japan Union Hospital of Jilin University. Two orthopedic surgeons were assigned to record clinical, pathological, and therapeutic information on the patient using a blinded method. In this study, patients with PMBNs were staged by the American Joint Committee on Cancer (AJCC) tumor node metastasis (TNM) classification system. Patients who met the following criteria were included in the study: (1) diagnosis was histologically confirmed, (2) AJCC stage of III–IV, and (3) with adequate follow-up. Patients who met the following criteria were excluded: (1) PMBNs were not the first tumor; (2) unknown whether surgery or not; and (3) unknown TNM stage, race, histological type, marital status, and tumor size. The demographic information, clinicopathological variables, and survival data of eligible patients were included (race, age, gender, histological type, histological grade, primary site, tumor size, TNM stage, marital status, radiotherapy, chemotherapy, surgery at primary site, surgery at DM, and follow-up information). The information contained within both the SEER database and our medical institution’s records lacks personally identifiable data, thus negating the requirement for patient-informed consent. Consequently, our local ethics committee waived the need for ethics approval. The term “overall survival” (OS) is defined as the time interval from the date of diagnosis to the date of death from any cause, whereas “cancer-specific survival” (CSS) refers to the duration from the initial diagnosis of PMBNs to death specifically attributable to cancer. The selection process for the study population and the overall study design workflow are depicted in **Figure 1**.

### Statistical analysis

According to the status of surgical treatment, the patients with advanced PMBNs were divided into two groups: the surgery group and the non-surgery group. The propensity score matching (PSM) method was employed to balance significant patient characteristics between these two groups to mitigate potential bias in the baseline data. Patients were matched on the logit scale using the nearest propensity score (PS) in a 2:1 ratio (with a caliper value of 0.03). Chi-square tests were conducted to assess all study variables, both before and after PSM. A Kaplan–Meier survival curve and a log-rank test were plotted to compare OS and CSS between the two groups. A multivariate Cox proportional hazard regression analysis was further executed to determine the relationship between PTR and survival outcomes. Furthermore, the hazard ratio (HR) and its 95% confidence intervals (CIs) were computed. All statistical methods in this study were performed with the SPSS 25.0 (IBM Corporation, Armonk, NY, USA) and R version 4.0.2 software (http://www.r-project.org/).

### Construction and verification of nomogram

Our study hypothesized that patients diagnosed with advanced PMBNs who underwent PTR and survived beyond the median OS of patients who did not receive PTR would benefit from this surgical procedure. Patients from the surgery group were arbitrarily split into a training set and a validation set at a ratio of 1:1. The training set was utilized for constructing a nomogram, whereas the validation set and the external validation set were deployed for the nomogram’s validation. Afterward, the univariate and multivariate logistic regression analyses were carried out to identify the predictors independently associated with surgical benefits. On the basis of these identified factors related to surgical benefits, we established a visually appealing nomogram utilizing the “rms” package in R software. Furthermore, we developed a web-based probability calculator using the “Dynnom” package. The nomogram’s discrimination power was assessed by the receiver operating characteristic (ROC) curves and their corresponding area under the curve (AUC). Calibration plots were generated to evaluate the concordance between the predicted and actual outcomes of the patients.

### Clinical utility of the nomogram

We employed decision curve analysis (DCA) curves to appraise the net clinical benefit of the predictive model. In an additional effort to authenticate the clinical utility of the nomogram, the total points of each patient in both the training set and the validation set were computed. Subsequently, different benefit states were established. Patients whose beneficial probability exceeded 0.5 were classified into the Sur-Benefit subset, and those with a beneficial probability of 0.5 or less were categorized into the Sur-Nonbenefit subset. Kaplan–Meier survival curves were utilized to compare OS across these three groups and to test whether the nomogram could successfully discern patients who would reap the benefits of surgery.

## Result

### Clinicopathologic characteristics before and after PSM

**Figure 2:**
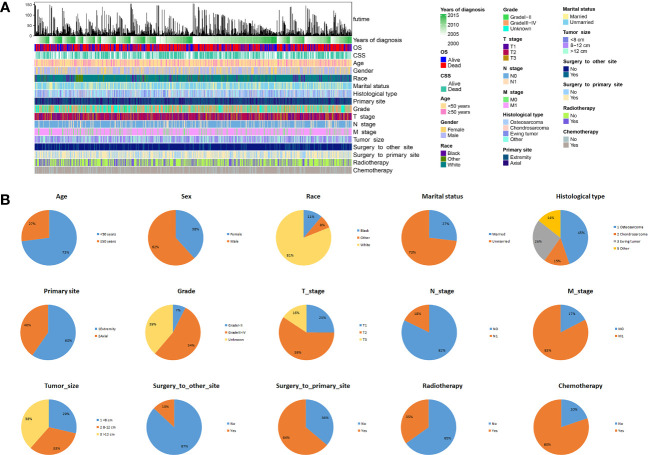
The epidemiological analysis of 839 patients with advanced PMBNs. **(A)** The integrated bar plot and heatmap of demographics information, tumor characteristics, and clinical outcomes of patients with advanced PMBNs. **(B)** The pie chart of variables in the patients with advanced PMBNs. PMBNs, primary malignant bone neoplasms.

Between 2004 and 2015, a total of 839 patients diagnosed with advanced PMBNs were identified in the SEER database. Among these, 536 patients (or 63.9%) underwent PTR, whereas the remaining 303 patients (or 36.1%) did not receive surgical treatment. Comprehensive demographic information, tumor characteristics, and patient outcomes were encapsulated in an integrated bar plot and heatmap (**Figure 2**). In addition, 43 eligible patients from our medical institution were incorporated into the study to validate the discriminative power of the newly developed nomogram externally. Significant discrepancies were observed in variables such as age, histology type, primary site, grade, N stage, M stage, surgery to DM, and radiotherapy between the surgery group and the non-surgery group. This indicates that the baseline characteristics between the two groups were not harmonized (P < 0.05) (**Table 1**). Following a 2:1 PSM analysis, 364 patients were matched to the surgery group and 182 to the non-surgery group. After PSM, all clinicopathologic variables, except for radiotherapy, were balanced after PSM (P > 0.05) (**Table 2**).

**Table 1:** Clinical and pathological characteristics for patients with advanced PMBNs before PSM.

Variables	Overall (n = 546, %)	Non-surgery (n = 182, %)	Surgery (n = 364, %)	p-value
Age				0.012
<50 years	612 (72.94)	205 (67.66)	407 (75.93)	
≥50 years	227 (27.06)	98 (32.34)	129 (24.07)	
Race				0.843
Black	94 (11.20)	36 (11.88)	58 (10.82)	
Other	65 (7.75)	22 (7.26)	43 (8.02)	
White	680 (81.05)	245 (80.86)	435 (81.16)	
Gender				0.950
Female	321 (38.26)	115 (37.95)	206 (38.43)	
Male	518 (61.74)	188 (62.05)	330 (61.57)	
Marital status				0.240
Married	225 (26.82)	89 (29.37)	136 (25.37)	
Unmarried	614 (73.18)	214 (70.63)	400 (74.63)	
Histological type				<0.001
Osteosarcoma	375 (44.70)	84 (27.72)	291 (54.29)	
Chondrosarcoma	126 (15.02)	43 (14.19)	83 (15.49)	
Ewing tumor	217 (25.86)	120 (39.60)	97 (18.10)	
Other	121 (14.42)	56 (18.48)	65 (12.13)	
Primary site				<0.001
Extremity	500 (59.59)	116 (38.28)	384 (71.64)	
Axial	339 (40.41)	187 (61.72)	152 (28.36)	
Grade				<0.001
Grade I–II	63 (7.51)	22 (7.26)	41 (7.65)	
Grade III–IV	451 (53.75)	106 (34.98)	345 (64.37)	
Unknown	325 (38.74)	175 (57.76)	150 (27.99)	
T stage				0.648
T1	210 (25.03)	78 (25.74)	132 (24.63)	
T2	497 (59.24)	182 (60.07)	315 (58.77)	
T3	132 (15.73)	43 (14.19)	89 (16.60)	
N stage				0.044
N0	690 (82.24)	238 (78.55)	452 (84.33)	
N1	149 (17.76)	65 (21.45)	84 (15.67)	
M stage				0.0003
M0	146 (17.40)	33 (10.89)	113 (21.08)	
M1	693 (82.60)	270 (89.11)	423 (78.92)	
Tumor size				0.586
<8 cm	240 (28.61)	88 (29.04)	152 (28.36)	
8–12 cm	276 (32.90)	105 (34.65)	171 (31.90)	
>12 cm	323 (38.50)	110 (36.30)	213 (39.74)	
Surgery to DM				<0.001
No	729 (86.89)	285 (94.06)	444 (82.84)	
Yes	110 (13.11)	18 (5.94)	92 (17.16)	
Radiotherapy				<0.001
No	546 (65.08)	138 (45.54)	408 (76.12)	
Yes	293 (34.92)	165 (54.46)	128 (23.88)	
Chemotherapy				1.000
No	166 (19.79)	60 (19.80)	106 (19.78)	
Yes	673 (80.21)	243 (80.20)	430 (80.22)	

**Table 2:** Clinical and pathological characteristics for patients with advanced PMBNs after PSM.

Variables	Overall (n = 546, %)	Non-surgery (n = 182, %)	Surgery (n = 364, %)	p-value
Age				0.722
<50 years	370 (67.77)	121 (66.48)	249 (68.41)	
≥50 years	176 (32.23)	61 (33.52)	115 (31.59)	
Race				0.984
Black	74 (13.55)	24 (13.19)	50 (13.74)	
Other	33 (6.04)	11 (6.04)	22 (6.04)	
White	439 (80.40)	147 (80.77)	292 (80.22)	
Gender				0.562
Female	235 (43.04)	82 (45.05)	153 (42.03)	
Male	311 (56.96)	100 (54.95)	211 (57.97)	
Marital status				1.000
Married	168 (30.77)	56 (30.77)	112 (30.77)	
Unmarried	378 (69.23)	126 (69.23)	252 (69.23)	
Histological type				0.058
Osteosarcoma	216 (39.56)	62 (34.07)	154 (42.31)	
Chondrosarcoma	100 (18.32)	28 (15.38)	72 (19.78)	
Ewing tumor	141 (25.82)	54 (29.67)	87 (23.90)	
Other	89 (16.30)	38 (20.88)	51 (14.01)	
Primary site				0.054
Extremity	315 (57.69)	94 (51.65)	221 (60.71)	
Axial	231 (42.31)	88 (48.35)	143 (39.29)	
Grade				0.409
Grade I–II	51 (9.34)	15 (8.24)	36 (9.89)	
Grade III–IV	282 (51.65)	89 (48.90)	193 (53.02)	
Unknown	213 (39.01)	78 (42.86)	135 (37.09)	
T stage				0.832
T1	160 (29.30)	53 (29.12)	107 (29.40)	
T2	306 (56.04)	100 (54.95)	206 (56.59)	
T3	80 (14.65)	29 (15.93)	51 (14.01)	
N stage				0.846
N0	445 (81.50)	147 (80.77)	298 (81.87)	
N1	101 (18.50)	35 (19.23)	66 (18.13)	
M stage				0.350
M0	91 (16.67)	26 (14.29)	65 (17.86)	
M1	455 (83.33)	156 (85.71)	299 (82.14)	
Tumor size				0.876
<8 cm	175 (32.05)	60 (32.97)	115 (31.59)	
8–12 cm	180 (32.97)	61 (33.52)	119 (32.69)	
>12 cm	191 (34.98)	61 (33.52)	130 (35.71)	
Surgery to DM				0.131
No	487 (89.19)	168 (92.31)	319 (87.64)	
Yes	59 (10.81)	14 (7.69)	45 (12.36)	
Radiotherapy				0.008
No	347 (63.55)	101 (55.49)	246 (67.58)	
Yes	199 (36.45)	81 (44.51)	118 (32.42)	
Chemotherapy				0.858
No/Unknown	128 (23.44)	44 (24.18)	84 (23.08)	
Yes	418 (76.56)	138 (75.82)	280 (76.92)	

### The correlation between PTR, radiotherapy, chemotherapy, and survival outcomes in patients with advanced PMBNs

**Figure 3:**
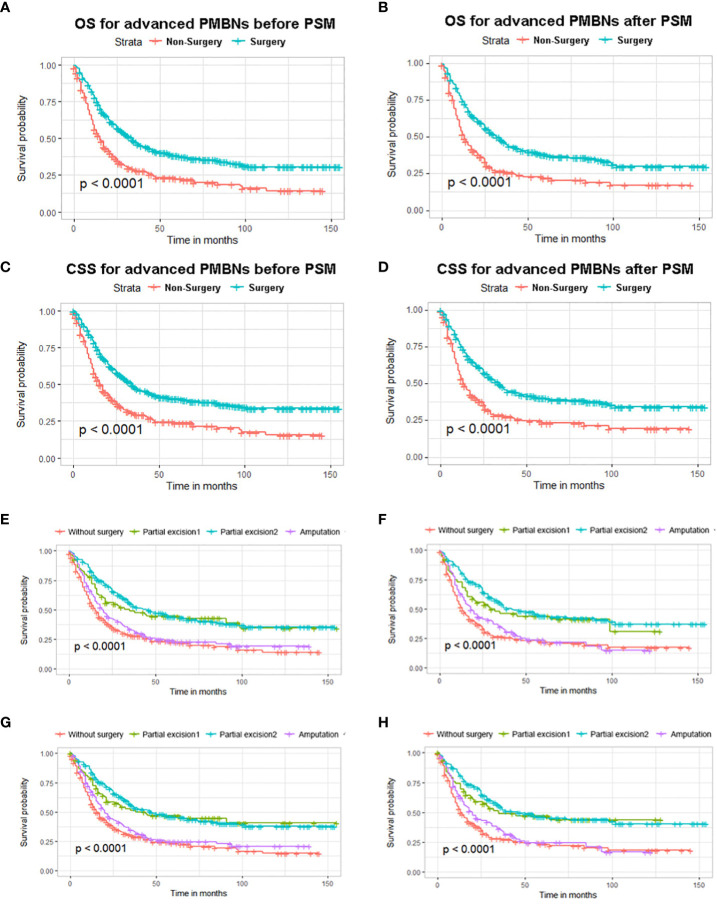
The impact of primary tumor resection on the survival outcomes of patients with advanced PMBNs. Kaplan–Meier survival curves of OS before PSM **(A)** and after PSM **(B)** and of CSS before PSM **(C)** and after PSM **(D)** in the surgery and non-surgery groups. OS analysis of different surgical approaches before PSM **(E)** and after PSM **(F)**, and CSS analysis of different surgical approaches before PSM **(G)** and after PSM **(H)**. PMBNs, primary malignant bone neoplasms; OS, overall survival; CSS, cancer-specific survival; PSM, propensity score matching.

Kaplan–Meier curves with log-rank test showed that patients who received PTR had longer OS and CSS than patients without surgery before and after PSM (**Figures 3A–D**). In addition, the median OS and CSS of the surgery group were 34 months (95% CI, 29.09–38.91) and 34 months (95% CI, 27.51–40.50), whereas the median OS and CSS of the non-surgery group were 15 months (95% CI, 12.61–17.39) and 15 months (95% CI, 12.30–17.71). After PSM, the survival benefit of PTR remained in patients with advanced PMBNs, the median OS and CSS of the surgery group were 32 months (95% CI, 26.46–37.54) and 34 months (95% CI, 28.01–39.99), whereas the median OS and CSS of the non-surgery group were 13 months (95% CI, 9.98–16.02) and 14 months (95% CI, 10.52–17.48). According their surgical status, all patients were classified as follows: without surgery (patients who did not receive PTR), partial excision 1 [patients who received local tumor destruction or partial resection/internal hemipelvectomy (pelvis)], partial excision 2 (patients who received radical excision or resection of lesion with limb salvage), and amputation; subsequently, a survival analysis of the different surgical procedures was conducted, with the results showing that, while surgery can improve survival in patients with advanced PMBNs, amputation does not appear to provide a survival benefit for OS and CSS in this patient group, and there was no statistical difference between local tumor destruction and limb salvage (all the p-value > 0.05), but the survival benefits for OS and CSS of local tumor resections (both limb salvage and local tumor destruction) were significantly better than amputation and no surgery (all the p-value < 0.05) (**Figures 3E–H**).

**Figure 4:**
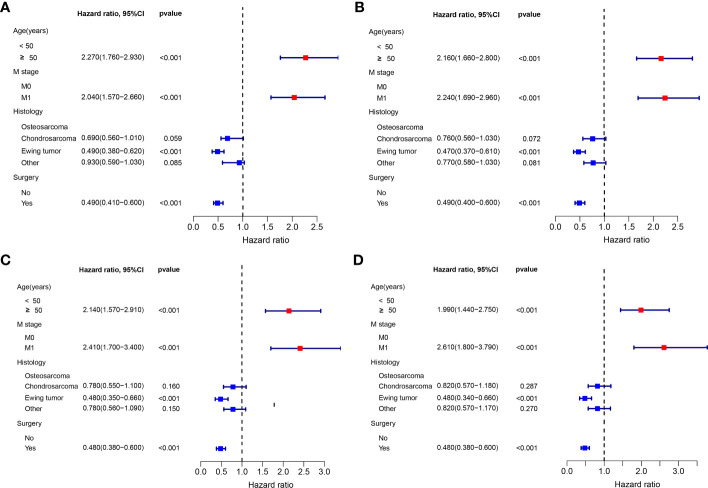
The forest plot for illustration of results of multivariate Cox regression analysis in patients with advanced PMBNs for OS before PSM **(A)** and after PSM **(C)** and for CSS before PSM **(B)** and after PSM **(D)**. PMBNs, primary malignant bone neoplasms; OS, overall survival; CSS, cancer-specific survival; PSM, propensity score matching.

**Figure 5:**
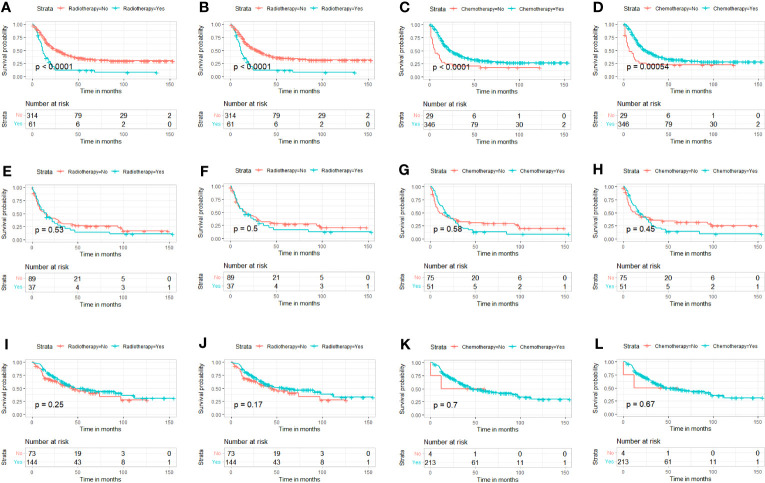
K-M survival analysis to study the correlation between radiotherapy and survival in advanced osteosarcoma [**(A)** OS; **(B)** CSS], the correlation between chemotherapy and survival in advanced osteosarcoma [**(C)**, OS; **(D)**, CSS]. The correlation between radiotherapy and survival in advanced chondrosarcoma [**(E)** OS; **(F)** CSS], and the correlation between chemotherapy and survival in advanced chondrosarcoma [**(G)** OS; **(H)** CSS]. The correlation between radiotherapy and survival in advanced Ewing sarcoma [**(I)** OS; **(J)** CSS)], and the correlation between chemotherapy and survival in advanced Ewing sarcoma [**(K)** OS; **(L)** CSS]. K-M, Kaplan–Meier; OS, overall survival; CSS, cancer-specific survival.

The multivariate Cox proportional hazard regression analysis indicated that surgery was an independent protective factor for both OS and CSS, both before and after PSM (**Figure 4**). Furthermore, considering the variability in the effectiveness of radiotherapy and chemotherapy depending on the histological type of PMBNs, we analyzed the relationship between these treatment modalities and survival outcomes among patients with different histological types of advanced PMBNs. The findings suggest that neither radiotherapy nor chemotherapy impacted the prognosis of patients with advanced chondrosarcoma or advanced Ewing sarcoma (**Figures 5E–L**). However, for patients with advanced osteosarcoma, adjuvant chemotherapy still demonstrated significant survival benefits (p < 0.05; **Figures 5C, D**), whereas radiotherapy appeared to adversely affect the survival outcomes in this patient group (p < 0.05; **Figures 5A, B**).

### Nomogram to identify optimal patients for surgery

**Figure 6:**
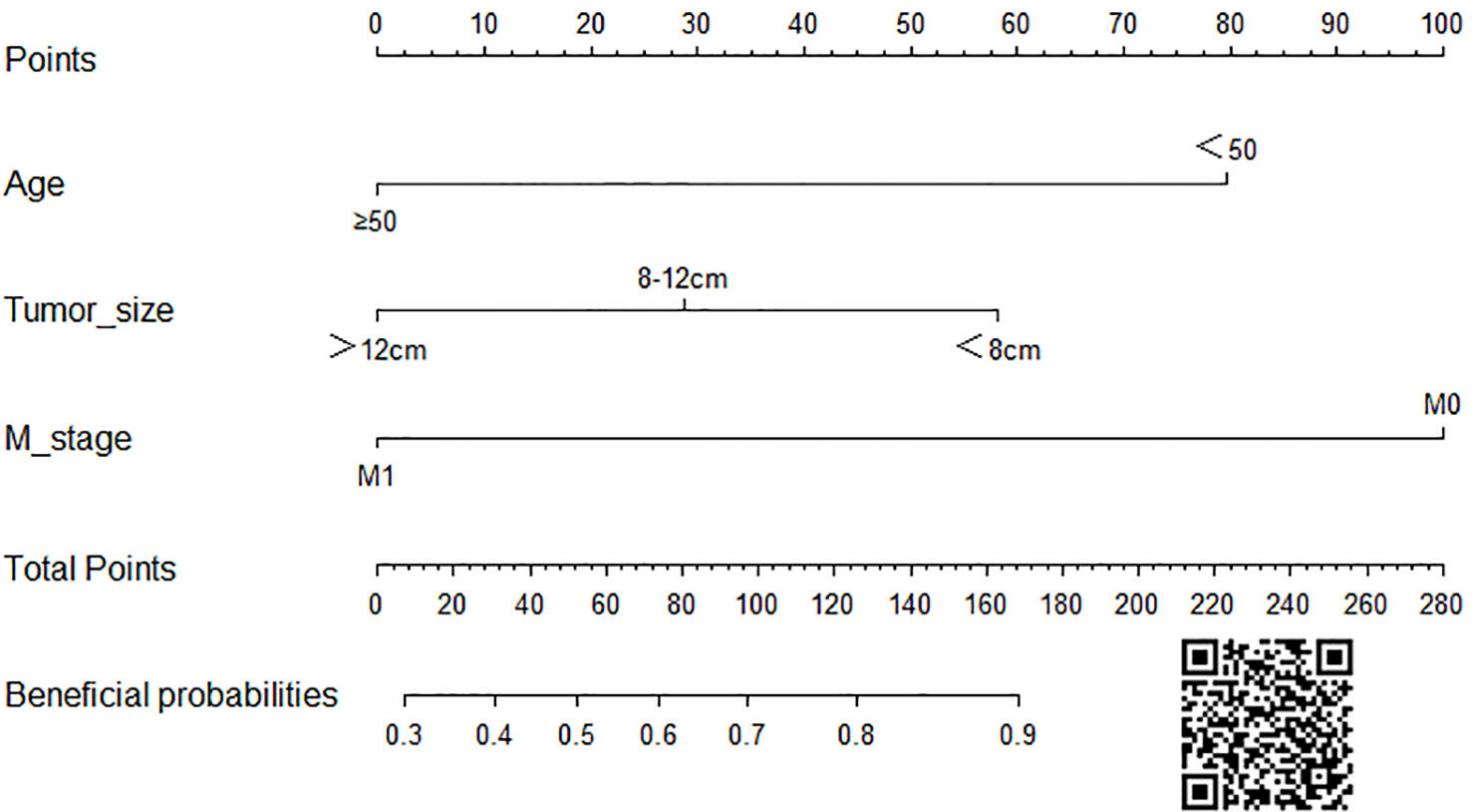
The visualized nomogram to predict the probability of surgical benefit in patients with advanced PMBNs. PMBNs, primary malignant bone neoplasms.

**Figure 7:**
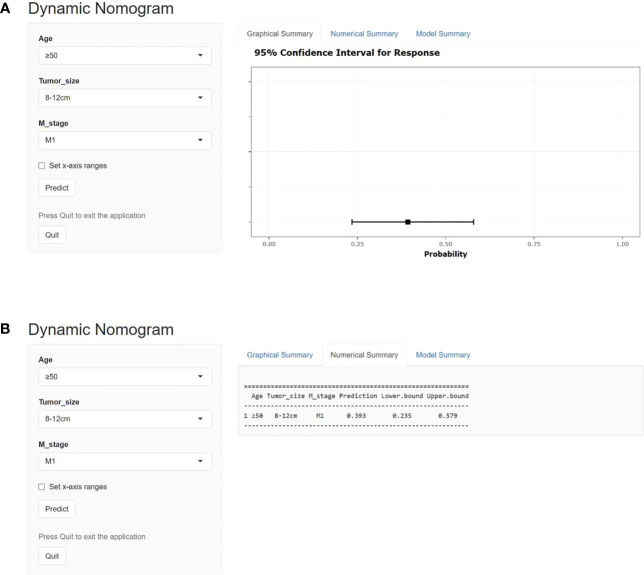
The operator interface of the web-based probability calculator. **(A)** Graphically demonstrates representation of the expected benefit rate for this patient, and **(B)** is specific value for the expected benefit rate for this patient.

**Figure 8:**
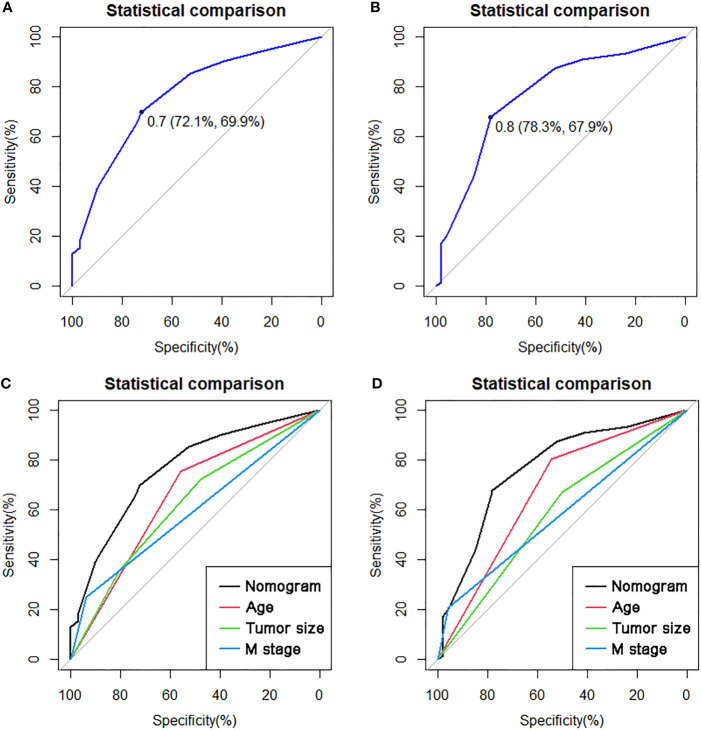
The ROC curves in the training set **(A)** and the validation set **(B)**. Comparison of the value of AUC between comprehensive nomogram and each independent predictors in the training set **(C)** and the validation set **(D)**. ROC, receiver operating characteristic curve; AUC, area under the curve.

**Figure 9:**
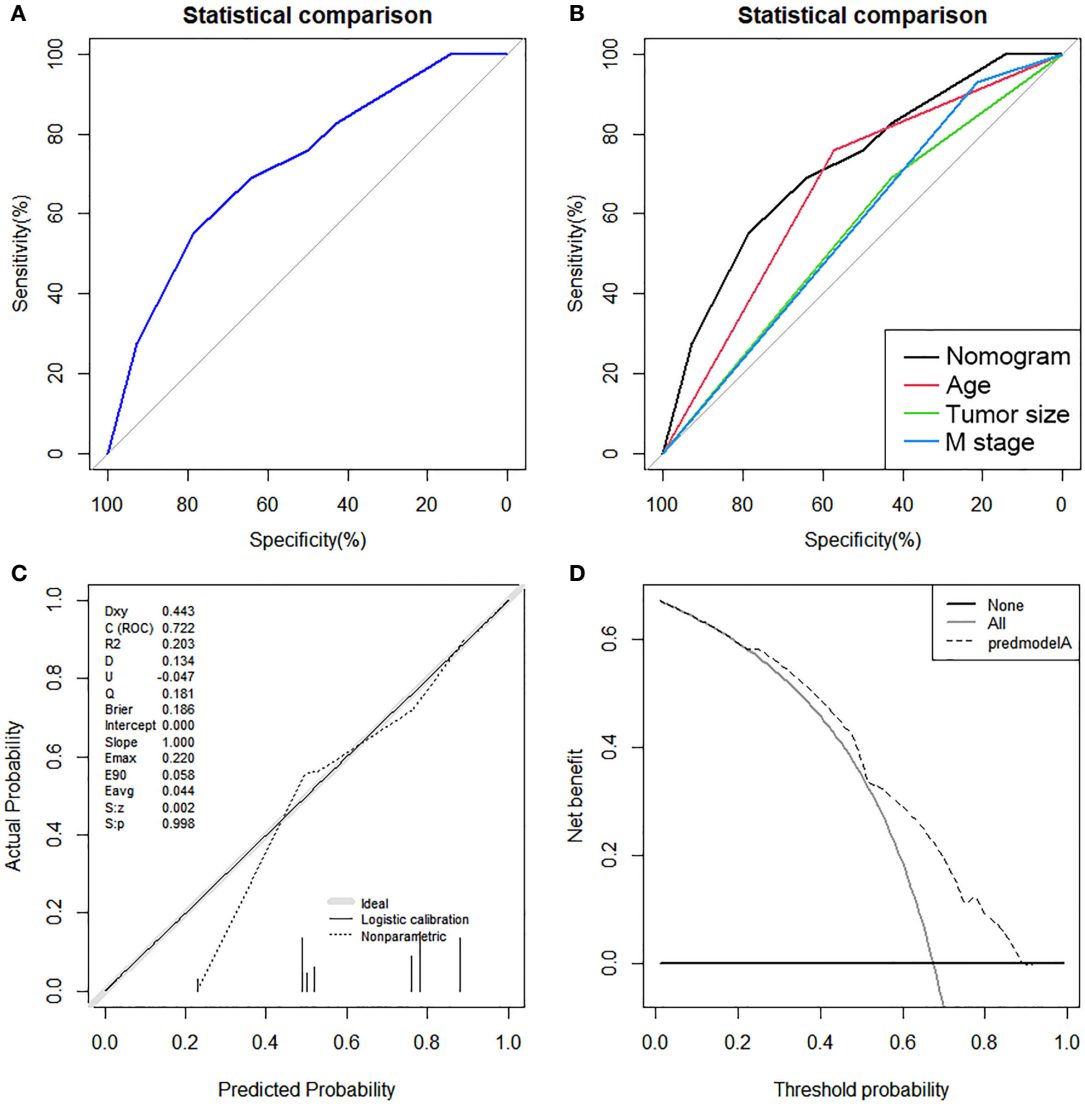
The ROC curve **(A)**, comparison of the value of AUC **(B)**, calibration curve **(C)** and DCA curve **(D)** of the external validation set.

**Figure 10:**
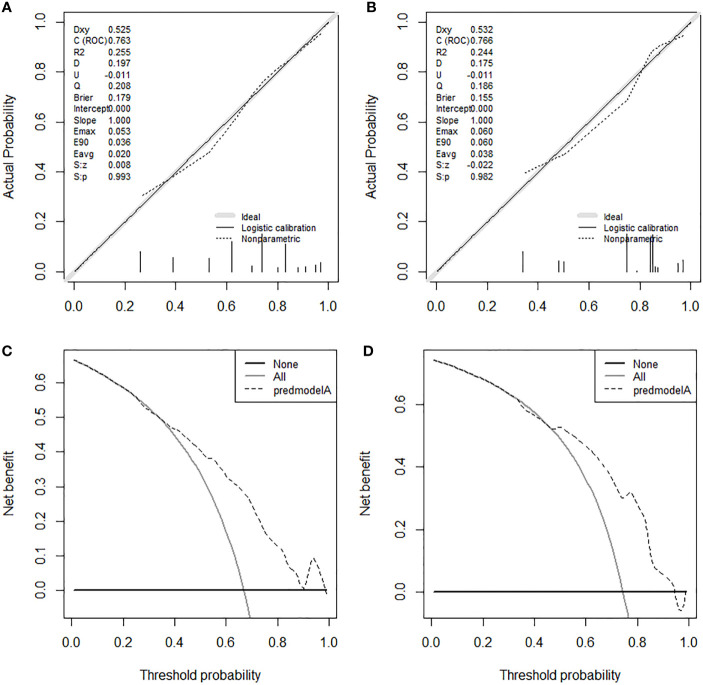
The calibration curves in the training set **(A)** and the validation set **(B)**. The DCA curves in the training set **(C)** and the validation set **(D)**. DCA, decision curve analyses.

On the basis of our assumption, patients with advanced PMBNs who received PTR were divided into a Sur-Benefit subset (survival time greater than 13 months) and a Sur-Nonbenefit subset (survival time less than or equal to 13 months). The univariate and multivariate logistic regression analyses determined three independent surgery benefit–related factors: age, M stage, and tumor size (**Table 3**). Then, a nomogram model was constructed to quantify the probability of surgical benefit and thus screen optimal candidates for surgical resection of primary tumors among patients with advanced PMBNs (**Figure 6**), which can be accessed via https://yxyx.shinyapps.io/sugicalbenefitofadvancedPMBNs/ (**Figure 7**). Moreover, the ROC curves were drawn for both the training and validation sets to assess the predictive capacity of this model. The AUC of the nomogram was 0.763 (95% CI, 0.691–0.835) in the training set (**Figure 8A**), 0.766 (95% CI, 0.685–0.848) in the validation set (**Figure 8B**), and 0.722 (95% CI, 0.640–0.878) in the external validation set (**Figure 9A**). Moreover, the AUC value of the comprehensive model exceeded the AUCs of age, size, and M stage individually in all three sets, indicating the robust discriminatory power of the nomogram (**Figures 8C, D**, **9B**). The calibration plots demonstrated an excellent alignment between the nomogram’s prediction and the actual outcomes in the training set (**Figure 10A**), the validation set (**Figure 10B**), and the external validation set (**Figure 9C**). The DCA curves demonstrated a positive net benefit in all three sets, reinforcing the strong clinical utility of the nomogram (**Figures 10C, D**, **9D**).

**Table 3:** Univariate and multivariate logistic analyses of factors related to surgical benefit in patients with advanced PMBNs.

Variables	Univariate analysis			Multivariate analysis		
	OR	95% CI	p-value	OR	95% CI	p-value
Age						
<50 years	Reference			Reference		
≥50 years	0.26	0.13–0.49	<0.001	0.22	0.11–0.44	<0.001
Race						
Black	Reference					
Other	0.72	0.16–3.27	0.671			
White	0.82	0.32–2.09	0.671			
Gender						
Female	Reference					
Male	0.92	0.49–1.71	0.783			
Marital status						
Married	Reference					
Unmarried	1.48	0.77–2.84	0.244			
Histological type						
Osteosarcoma	Reference					
Chondrosarcoma	0.56	0.25–1.28	0.171			
Ewing sarcoma	1.65	0.68–4.01	0.266			
Other	0.66	0.28–1.56	0.342			
Primary site						
Extremity	Reference					
Axial	1.06	0.56–2	0.86			
Grade						
Grade I–II	Reference					
Grade III–IV	0.59	0.21–1.68	0.324			
Unknown	2.62	0.79–8.68	0.116			
T stage						
T1	Reference					
T2	0.5	0.24–1.04	0.064			
T3	0.89	0.32–2.49	0.83			
N stage						
N0	Reference					
N1	2.17	0.77–6.11	0.14			
M stage						
M0	Reference			Reference		
M1	0.21	0.07–0.62	0.005	0.15	0.05–0.49	0.002
Tumor size						
<8 cm	Reference			Reference		
8–12 cm	0.61	0.27–1.4	0.247	0.57	0.23–1.41	0.227
>12 cm	0.32	0.14–0.72	0.006	0.33	0.14–0.79	0.013
Surgery to DM						
No	Reference					
Yes	1.83	0.58–5.82	0.306			
Radiotherapy						
No	Reference					
Yes	1.31	0.66–2.59	0.445			
Chemotherapy						
No	Reference					
Yes	1.97	0.97–3.99	0.061			

**Figure 11:**
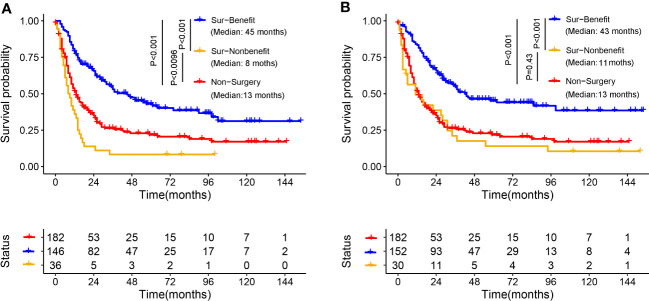
Validation of the distinguishing ability of nomogram in the matched cohort. K-M survival analysis to compare survival difference of the patients among the Sur-Benefit, Sur-Nonbenefit, and Non-Surgery groups in the training set **(A)** and the validation set **(B)**. K-M, Kaplan–Meier.

Kaplan–Meier survival analysis was used to validate the discriminative capacity of the nomogram by comparing the survival disparities between the Sur-Benefit subset, Non-Benefit subset, and the Non-Surgery group. The Sur-Benefit subset displayed a higher survival rate in the training set than the Non-Benefit and Non-Surgery groups (P < 0.001). Notably, patients not anticipated to benefit from PTR demonstrated even poorer prognoses than those who did not undergo surgery (P = 0.0096), suggesting the nomogram’s excellent potential in identifying the most suitable candidates for PTR (**Figure 11A**). In the validation set, the Sur-Benefit subset survived longer than the Non-Benefit subset and the Non-Surgery group (P < 0.001), whereas no difference was observed between the Non-Benefit subset and the Non-Surgery group (**Figure 11B**).

## Discussion

The rare nature of advanced PMBNs inevitably contributes to a paucity of research focused on clinical management strategies, leading to differing viewpoints in medical decision-making when treating these patients (23). Recent literature has underscored the survival benefits associated with PTR in patients with PMBNs (24, 25), significantly attributed to the reversal of immune suppression triggered by the tumor. Tumor-bearing patients often exhibit an “ignorance” or inadequate response to tumor antigens due to shortfalls in T cells, B cells, and antigen-presenting cells, consequently inducing tumor-mediated immunosuppression (26, 27). Surgical resection of the solid tumor partially revives immune competency, with both CD4+ T cells and CD8+ T cells implicated in re-establishing tumor immunity (27). Therefore, even in advanced tumors, excision of the primary lesion can impart a degree of survival benefit to patients. However, a study by Song et al. suggested limited survival benefit of PTR in patients with DM and spinal chondrosarcoma patients for over 70 years (28). Furthermore, another study proposed that surgery did not significantly influence survival rates in patients with metastatic axial (pelvic/spinal) osteosarcoma (29). These collective findings hint at the notion that, due to inherent patient heterogeneity, not all patients with advanced PMBNs may benefit from the procedure. Current studies still lack a reliable and user-friendly tool that can inform orthopedic surgeons about the individual-specific probability of obtaining a benefit from PTR.

In this study, we not only reinforced the beneficial impact of PTR in the treatment of patients with advanced PMBNs, but, crucially, we also developed a visualized nomogram designed to precisely categorize these patients based on their anticipated probability of benefiting from localized surgery. The validation to this model exhibited exceptional discriminatory capacity and clinical utility. Moreover, the validation of an externally sourced dataset from another geographical area showcased the wide-ranging applicability of the model. As illustrated in **Figure 11**, our comprehensive model significantly outperforms assessments based on individual clinicopathological attributes when evaluating the potential for surgical benefit. We have also developed a web-based probability calculator to enhance its clinical utility. In simple terms, users can visit the provided website or scan the QR code and input the patient’s age, tumor size, and metastasis status on the left-hand side of the web interface. Upon clicking the “Predict” button, the calculated probability of surgical benefit for the patient appears on the right-hand side of the web interface (as shown graphically in **Figure 7A**) and the specific data (presented in **Figure 7B**). Importantly, our data indicate that, for patients projected by our model to not benefit from surgery, PTR does not appear to improve their prognosis, with their survival even appearing to be worse than those who did not undergo PTR, possibly due to surgical complications (30). These findings echo previous studies and further validate the necessity of a comprehensive assessment of surgical benefit probability for the clinical management of patients with advanced PMBNs. Patient selection is critical to achieving significant improvements in survival after PTR.

Our study suggests that M stage, age at diagnosis, and tumor size are independently related to the potential benefits patients can derive from PTR. Of these variables, the status of DM exhibited the strongest correlation with the probability of surgical benefit. Previous studies have demonstrated improved survival in in patients with PMBNs with metastatic disease who underwent PTR (31, 32). This could be attributed to the reduction of the overall tumor load and the eradication of the primary source of cells capable of metastasizing (33). Nevertheless, the “self-seeding” theory suggests that circulating tumor cells (CTCs) originating from metastatic sites could return to the primary site, thus promoting local tumor progression (33). Despite the majority of CTCs perishing in the hostile environment of the circulatory system, the surviving cells that return to the primary site could create a favorable tumor microenvironment by inhibiting immune surveillance, enhancing angiogenesis, supporting tumor growth, and fostering further metastases (34, 35). As noted earlier, even some animal models have shown the reversal of immunosuppression following the resection of the primary tumor in the presence of persistent metastases (16). The topic of surgical intervention in patients with metastatic PMBNs remains somewhat contentious, with conflicting results reported in several studies (36, 37). The “dormancy hypothesis” proposes that the growth of the metastatic site typically comprises temporary dormancy of the single-cell stage and the avascular micrometastasis stage. Patients who underwent PTR exhibited significantly elevated levels of vascular endothelial growth factor (VEGF), epidermal growth factor (EGF)–like growth factors, and other yet unidentified proliferative inducers, compared with their respective serum levels (38, 39). This theory also indicates that the release of these mediators, caused by the surgical procedure, could lead to a surgery-driven escape from dormancy and the subsequent acceleration of relapses (40). These observations imply that metastatic disease must be fully considered when evaluating the probability of surgical benefit in patients with advanced PMBNs. Furthermore, our results show that older patients might derive fewer benefits from PTR, potentially due to poorer nutritional status, decreased physiological reserve, more complex underlying conditions, and reduced tolerance to surgical treatment. Previous studies also show that axial bone involvement is higher in older patients with PMBNs compared with their younger counterparts (41, 42). The complicated anatomy of the axial bone site leads to more significant surgical risks and technical difficulties potentially resulting in more severe complications. Given the frail physical condition of the elderly, the likelihood of surgical benefit in this population is significantly reduced (43). The primary tumor size was also identified as an independent factor associated with the potential benefit of surgery. A larger tumor size increases the possibility of positive surgical margins, and larger tumors are often characterized by more aggressive biological behavior, indicating a higher risk of local recurrence. Therefore, the newly developed nomogram, which includes the predictors mentioned above, could be valuable for estimating the probability of surgical benefit and subsequently identifying the most suitable candidates for PTR among patients with advanced PMBNs. It should be acknowledged that this study also has some limitations. First, the general condition information of patients was not recorded in the SEER database, which might have a biased effect on the choice of surgical treatment in patients with advanced PMBNs. Second, increasing evidence indicates that neoadjuvant chemotherapy (NACT) and neoadjuvant chemoradiotherapy (NACTRT) can provide survival benefits for patients with PMBNs. In contrast, detailed protocols and doses of radiotherapy and chemotherapy were not available from this database. Finally, the metastatic site was a crucial factor in the prognosis of patients with PMBNs, especially the lung metastasis. However, because of the limitation of the SEER database in finding the year of record, we could not obtain a sufficient sample size of patients with known metastatic conditions for analysis.

## Conclusion

Our study shows that PTR can improve survival in advanced PMBNs, except for amputation. Using a well-validated prediction model, we quantified the probability of benefiting from PTR in these patients, thus helping to allocate surgical treatment more appropriately.

## Data availability statement

The raw data supporting the conclusions of this article will be made available by the authors, without undue reservation.

## Ethics statement

We received permission to access the research data file in the SEER program from the National Cancer Institute, USA (reference number 15685-Nov2020). Approval was waived by the ethics committee of China–Japan Union Hospital of Jilin University, as research data is publicly available and de-identified.

## Author contributions

YT and DZ conceived of and designed the study. YT and YC collected the clinical data and literature review. YT conducted the statistical analysis. YT, LJ, YP, and YG generated the figures and tables. YT wrote the manuscript. YT and DZ revised the manuscript. DZ supervised the research. All authors critically read the manuscript to improve intellectual content. All authors contributed to the article and approved the submitted version.
